# Bioelectrical Impedance Assessment in a Patient with Breast Cancer: A Case Report on the Effect of Integrative Therapies on Cellular Homeostasis

**DOI:** 10.3390/nu17152506

**Published:** 2025-07-30

**Authors:** Graziella Marino, Giovanni Pace, Lucia Sabato, Marzia Sichetti, Marisabel Mecca

**Affiliations:** 1 Unit of Breast Cancer, Centro di Riferimento Oncologico della Basilicata (IRCCS-CROB), 85028 Rionero in Vulture, Italy; graziella.marino@crob.it; 2 Associazione AGATA OdV ETS, 75015 Marconia di Pisticci, Italy; giovannipace67@gmail.com; 3 Associazione Italiana Rionero Onlus (AIRO), 85028 Rionero in Vulture, Italy; dottoressasabatolucia@gmail.com; 4 Laboratory of Preclinical and Translational Research, Centro di Riferimento Oncologico della Basilicata (IRCCS-CROB), 85028 Rionero in Vulture, Italy; marisabel.mecca@crob.it

**Keywords:** breast cancer, integrative therapies, quality of life, curcumin, polydatin, diet, Qigong, BIA, phase angle

## Abstract

**Background/Objectives**: Since breast cancer (BC) survival rates have increased to 91% at 5 years and 80% at 15 years postdiagnosis, there is a growing awareness of the importance of addressing the long-term well-being of patients. Consequently, integrative oncology, which combines standard therapies with complementary approaches (nutrition, mind–body practices, and lifestyle modifications), has emerged as a patient-centred model aimed at improving symptom management, treatment adherence, and overall quality of life (QoL). This study aims to demonstrate how integrative therapies can benefit body composition, phase angle, and fluid and electrolyte balance through bioelectrical impedance analysis (BIA). **Methods**: This study considers a patient who underwent BC surgery and was enrolled in the AMICO clinic for anamnesis, as well as their oncological pathology data, assessment of QoL, and BIA. The breast surgeon specialising in integrative oncology therapies prescribed the patient curcumin and polydatin, moderate physical activity, a balanced diet, and Qigong sessions. The patient underwent monitoring through haematochemical analysis, BIA, and a QoL questionnaire, with follow-up every four months. **Results**: Between 4 and 12 months, fat mass (FM) and body mass index (BMI) markedly decreased, whereas fat-free mass (FFM), total body water (TBW), and skeletal muscle mass (SMM) increased progressively. Moreover, the improvements in the Na/K ratio and phase angle (PhA) suggest a shift toward better electrolyte and fluid balance and enhanced cellular integrity and membrane function. Equally outstanding were her psychological benefits in terms of mood, sleep, anxiety, and melancholy. **Conclusions**: Patient progress in body composition, metabolic function, pain management, and psychological status measured during the 12-month follow-up demonstrates the potential benefits of an integrative approach to supportive cancer care.

## 1. Introduction

Cancer is a disease that has the potential to be life-threatening and can significantly affect patients’ physiological, psychological, and social well-being, as well as their quality of life (QoL) [1]. In particular, in breast cancer (BC), survival rates have improved globally, reaching 91% at 5 years and 80% at 15 years postdiagnosis [2,3] owing to early and accurate diagnosis and targeted treatment regimens. As patients live longer, addressing the multifaceted aspects of their lives, including their psychological and physical health, has become crucial. Throughout their cancer care journey, a substantial proportion of BC patients experience a combination of psychological and physical symptoms [4]. Depression and anxiety affect 10–30% of BC patients [5], whereas sleep disturbances are reported in up to 93% of cases [6]. Consequently, an integrative (or “complementary”) approach to routine cancer care has gained in importance, with QoL now recognised as a key outcome measure in clinical trials and survival studies. Integrative oncology aims to enhance symptom management, treatment adherence, and overall QoL before, during, and after cancer therapy [7,8,9,10].

This patient-centred approach combines conventional treatments with evidence-based complementary therapies, including natural products, lifestyle modifications, and mind–body disciplines. Epidemiological and clinical studies suggest that modifiable lifestyle factors, notably adherence to a Mediterranean-style dietary pattern, significantly influence BC risk and disease progression. Furthermore, preclinical and clinical investigations highlight the therapeutic potential of specific dietary phytochemicals and nutraceuticals. Key bioactive agents, such as indole-3-carbinol, omega-3 polyunsaturated fatty acids (PUFAs), curcumin, and polydatin, exhibit pleiotropic anticancer mechanisms, proapoptotic effects, inhibition of neoplastic cell proliferation, and suppression of tumour recurrence, in addition to their well-established anti-inflammatory and antioxidant properties [11,12]. These compounds may serve as adjunctive chemopreventive agents, offering a synergistic approach to conventional therapies. When combined with personalised nutrition and physical mind–body practices, these compounds may help support cellular function, alleviate oxidative stress, and promote recovery from the harmful effects of chemotherapy and radiotherapy. For this reason, in addition to nutrition, maintaining an active lifestyle is crucial [13,14,15]. Starting with moderate physical exercise and gradually increasing in intensity through a structured training regimen can further support overall health and reduce BC risk. In particular, Qigong, a gentle movement-based practice, focuses on cultivating energy (qi) through slow, controlled movements and improving balance, coordination, and relaxation. Its simplicity—often involving repeated single movements or short sequences—makes it particularly accessible for beginners, offering a manageable entry point for stress reduction and physical rehabilitation.

To objectively evaluate the physiological impact of such integrative therapies, bioelectrical impedance analysis (BIA) serves as a reliable, noninvasive, and cost-effective method for assessing body composition. BIA is widely utilised in clinical settings to monitor changes in body composition, particularly in patients undergoing medical treatments or therapies [16,17,18]. BIA provides valuable information on fat mass, lean mass, hydration status (including both intracellular and extracellular water), and electrolyte balance [16]. The technique involves placing electrodes on a person’s extremities and passing a small electrical current through the body [18,19]. The resulting measurements are based on the different electrical conductivity properties of various tissues. Lean tissue, such as muscle, conducts electricity well because of its higher water and electrolyte contents, whereas fat tissue has lower conductivity and offers more resistance because of its lower water content [16,17,18]. One of the key parameters derived from BIA is the phase angle (PhA), which reflects cellular health and tissue integrity. PhA represents the angular relationship between resistance and reactance in the bioelectrical signal and is strongly associated with the quality and functionality of cell membranes and body mass. Higher phase angle values generally indicate better cellular health and nutritional status, whereas lower values can suggest malnutrition, dehydration, reduced cell integrity, or the presence of pathological conditions [19,20]. Because BIA parameters are sensitive to physiological and pathological changes, they are valuable biomarkers for evaluating nutritional status and cellular health in the clinic, especially in oncology patients [19,21,22]. In oncology, chemotherapy and radiotherapy can profoundly affect cellular function.

These therapies may lead to reduced cell membrane integrity, diminished mitochondrial activity, and impaired protein synthesis, all of which can negatively influence BIA parameters, particularly PhA [23,24]. In the context of cellular physiology, the sodium-potassium pump (Na^+^/K^+^-ATPase) plays a fundamental role in maintaining ionic gradients across the cell membrane. This ionic exchange is vital for sustaining the resting membrane potential, regulating cell volume, and supporting the electrochemical gradients necessary for various cellular functions, including nerve signal transmission, muscle contraction, and nutrient transport [25,26]. Dysfunction of the Na^+^/K^+^-ATPase pump has profound implications, and disruption of the ionic balance can lead to electrolyte imbalances; alterations in cell membrane integrity and permeability; cellular depolarisation; and even the regulation of cell growth, differentiation, and apoptosis [27,28,29]. Since the Na^+^/K^+^-ATPase pump helps maintain the polarised state of the membrane and intracellular electrolyte homeostasis, it is reasonable to assume that its proper function contributes to higher PhA values, reflecting healthy, functional cells with intact membranes. Conversely, pump dysfunction can lead to lower PhA values, indicating membrane damage, fluid imbalance, and loss of cellular vitality. Hence, several studies have demonstrated that cancer cells often exhibit abnormal Na^+^/K^+^-ATPase activity, which may promote tumour progression, resistance to apoptosis, and adaptation to oxidative stress [27,28,29].

The case presented in this work involves a patient who underwent surgery for breast cancer at our Breast Unit and was then enrolled in the “AMICO” clinic—an acronym for Ambulatorio di Medicina Integrata e Condotta in Oncologia (i.e., Integrated Medicine and Oncology Management Clinic). Located within the Breast Surgery Unit at the Centro di Riferimento Oncologico della Basilicata (IRCCS-CROB) in Rionero in Vulture, southern Italy, the “AMICO” clinic is a pioneering initiative. It represents the first programme of its kind within a public hospital, offering free access to patients. This model integrates clinical care and research, promoting healthcare innovation while emphasising accessibility and inclusivity. The “AMICO” clinic adopts a multidisciplinary therapeutic approach that combines physical activity, personalised nutritional interventions, and the use of evidence-based nutraceuticals alongside mindfulness techniques. These strategies collectively aim to enhance immune function, optimise body composition, and improve tolerance to conventional cancer treatments. By mitigating stress and supporting cellular health, the program may also increase treatment efficacy and overall patient well-being.

Early observations from the clinic suggest that patients who adopt these strategies—Qigong, targeted nutraceutical supplementation (polydatin and curcumin), and lifestyle modifications—exhibit measurable benefits. These include reduced chemotherapy-related symptoms, such as pain, fatigue, and hot flashes, alongside marked improvements in QoL, spanning emotional stability, sleep quality, and physical functioning.

This study demonstrates how an integrated therapeutic approach can benefit body composition, phase angle, and fluid and electrolyte balance through BIA. By incorporating physical activity, personalised nutrition, and nutraceutical supplementation, this approach aims to support cellular health and address physiological imbalances, particularly those affecting the Na^+^/K^+^-ATPase pump. BIA utilises noninvasive methods to provide valuable insight into cellular function and treatment effectiveness, ultimately enabling personalised care strategies that increase electrolyte homeostasis, increase cellular resilience, and improve clinical outcomes for cancer patients.

In summary, AMICO’s model exemplifies the potential of integrative oncology in combination with conventional survival metrics, fostering long-term well-being through a holistic, patient-centred framework. By addressing physiological imbalances and metabolic dysregulation, this approach may redefine standards in supportive cancer care, emphasising both healing and quality of life.

## 2. Materials and Methods

### 2.1. Patient Description

**Figure 1:**
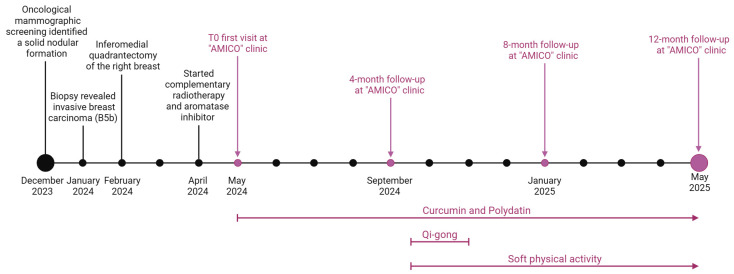
The patient’s timeline indicates the year and month of diagnosis, surgical and therapeutic management, “AMICO” clinic follow-up times (vertical bars and arrows above the timeline), and integrative therapies (vertical arrows and bars below the timeline). Created at https://BioRender.com by Mecca, M. (accessed on 22 May 2025).

A 73-year-old female with a history of no smoking, occasional alcohol consumption, osteoporosis, hypertension, hypercholesterolaemia, and menopause at age 48 (Table 1) was included. Following oncological mammographic screening provided free of charge by the Basilicata Region (southern Italy), a 6 mm nodule was found at the intersection of the quadrants of the right breast in December 2023 (Figure 1).

A bilateral breast ultrasound and a bilateral mammogram performed in January 2024 revealed a solid nodular formation with irregular margins of approximately 7 × 6 mm located at the junction between the two lower quadrants of the right breast, warranting a biopsy. The staging, also performed with a breast MRI, confirmed the presence of the breast nodule in the lower quadrants, and no additional solid nodular lesions nor axillary adenopathies were identified bilaterally.

The patient sought medical attention at a public clinic at the end of January 2024, where the medical staff performed a 16G cutting-needle biopsy of her right breast tumour (Figure 1). Histopathological (HP) examination revealed an infiltrating breast carcinoma (GATA3+) extending to the edges of the fragment, along with areas of ductal carcinoma of intermediate grade (G2) in situ and regions of infiltrative breast carcinoma. Immunohistochemical (IHC) reactions indicated invasive breast carcinoma (B5b), no special type (NST), E-cadherin positivity in the tumour arrangement, p63 negativity, oestrogen receptor positivity (ER 90%), progesterone receptor positivity (PR 1%), and Ki67: 10%. Immunohistochemical labelling revealed ambiguous overexpression of human epidermal growth factor receptor 2 (HER2). Subsequent fluorescence in situ hybridisation for HER2 did not reveal amplification.

The patient received a referral to a breast surgeon for further assessment. To thoroughly evaluate several therapeutic and surgical options, the breast surgeon presented the patient’s clinical case in a multidisciplinary oncology group that included consultants from radiation oncology and medical oncology services.

Treatment for malignant breast tumours has traditionally focused on surgical intervention. Surgical excision without axillary staging is indicated as the primary treatment for breast cancer by the National Comprehensive Cancer Network Guidelines [30]. Therefore, the patient underwent an inferomedial quadrantectomy of the right breast in February 2024 (Figure 1).

The histological exam confirmed a moderately differentiated infiltrating breast carcinoma NST G2, pT1b pN0 (0/2), Stage IA, and no involvement of the secondary axillary lymph node.

The patient underwent fifteen sessions of 3D conformal radiation therapy (3D-CRT), with a daily fraction dosage of 2.67 Gray (Gy) up to a total dose of 40.05 Gy over 3 weeks, starting in April 2024. Because she was postmenopausal, daily treatment with letrozole 2.5 mg, an aromatase inhibitor, for 5–10 years was the best treatment choice [30,31].

### 2.2. Study Intervention

#### 2.2.1. Integrative Therapy Assessment

Side effects, such as nausea, vomiting, marked fatigue, and a lack of appetite, with a net impaired QoL, appeared at the beginning of the treatment in April 2024.

Therefore, in May 2024, the patient underwent the first visit to the “AMICO” clinic for anamnesis and collection of clinical data on oncological pathology, current health status, and general QoL (Figure 1). In accordance with the American College of Lifestyle Medicine (ACLM) and Society for Integrative Oncology (SIO) clinical guidelines [14], the breast surgeon specialising in integrative oncology therapies prescribed curcumin and polydatin, which are known for their antitumour effects, along with common chemotherapy. The integrative treatment was administered during chemotherapy using a commercial dietary supplement with an oral gel formulation containing 300 mg of curcumin and 160 mg of polydatin taken once a day. Curcumin is titrated at ≥85%, while polydatin has a purity of ≥98%. The specialist also recommended an active lifestyle, starting with soft physical exercise, along with a training regimen that involved gradually increasing dedication (Figure 1).

#### 2.2.2. Bioelectrical Impedance Analysis (BIA)

BIA was performed via a portable segmental multifrequency (SMF)-BIA device (A-WAVE; RJL Systems, Detroit, MI, USA) at a follow-up visit every four months beginning in May 2024 (T_0_). Using four different frequencies (1, 5, 50, and 250 kHz) to define the resistance, reactance, impedance, and phase angle, the analyser estimates the segmental body composition and fluid status. A weak alternating current is applied at four sites (typically both wrists and ankles), and the body mass index (BMI), fat mass percentage (FM%), fat-free mass percentage (FFM%), skeletal muscle mass percentage (SMM%), intracellular water percentage (ICW%), extracellular water percentage (ECW%), total body water percentage (TBW%), Na^+^/K^+^ ratio, and phase angle (PhA) are automatically calculated and recorded (Table 1).

Before the examination, the patient was instructed to avoid excessive fluid intake and heavy physical activity in accordance with the manufacturer’s guidelines. The patient also received instructions to remove all socks, jewellery, and electronic devices and to remain in a supine position.

The specialist also recommended to the patient a balanced diet, such as the traditional Mediterranean diet [32,33], characterised by the consumption of bread, pasta, at least two servings of seasonal vegetables and fruits per day, fish, eggs, and predominantly white meats over red meats, as well as milk and soy-based products.

#### 2.2.3. Qigong Session

The Qigong intervention followed a standardised protocol taught by a certified instructor for 6 weeks, starting in September 2024 (Figure 1). This technique was developed by the Institute of Integral Qigong and Tai Chi and is based on a small number of Qigong movements as an integrated practice, taught as a series of repeated movements that are simple to learn (rather than long and difficult chains of choreographed Tai Chi forms) [34]. There were 7 core exercises and 10 additional movements taught with instructions to focus on slow and deep breathing coordinated with the movements and to use movement, flow, breath, and present-moment awareness to achieve a relaxed and meditative state.

### 2.3. Measures and Findings Follow-Up

The patient underwent monitoring (Table 2) through haematochemical analysis on the Beckman Coulter DxH 900 (Beckman Coulter, Miami, FL, USA), BIA, instrumental tests, and a QoL questionnaire based on the SF-12 and ESAS scales, with follow-up every four months beginning in May 2024 (T_0_).

## 3. Results and Discussion

Integrative oncology and holistic approaches are increasingly being explored as complementary strategies in managing breast cancer patients, aiming to improve their overall well-being, alleviate treatment-related side effects, and potentially enhance treatment outcomes. These approaches combine conventional cancer treatments (such as chemotherapy, radiation, and surgery) with complementary therapies (such as acupuncture, mindfulness, nutraceutical supplementation, and dietary modification) in a patient-centred manner [14,35,36,37].

This study demonstrates how a combined integrative therapeutic approach, consisting of dietary supplementation, mindful movement practices, and gradual physical activity, can greatly improve the well-being of BC patients recovering from the side effects of chemotherapy and radiotherapy. The patient’s progress in terms of body composition, metabolic function, pain management, and psychological state was measurable during the 12-month follow-up, indicating the potential benefits of a holistic approach to supportive oncology care.

**Figure 2:**
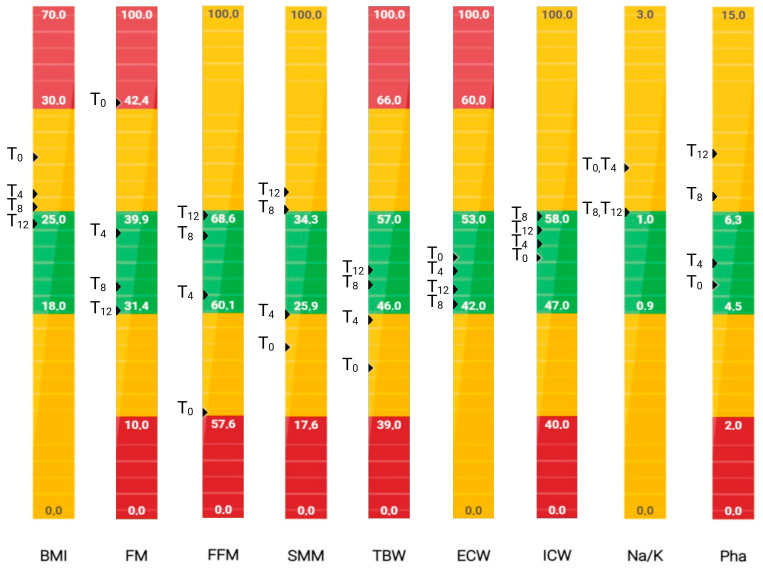
Patient graphical representation of body composition parameters obtained by bioelectrical impedance analysis (BIA) at T_0_, 4 months (T_4_), 8 months (T_8_), and 12 months (T_12_). The results are colour-coded according to clinical relevance: green indicates values within the normal physiological range, yellow indicates mildly altered values, and red indicates severely pathological values. Created at https://BioRender.com by Mecca, M. (accessed on 17 June 2025).

The graphical representation of the patient’s body composition parameters across the 12-month observation period revealed progressive and clinically significant improvements, as assessed by BIA (Figure 2). At T_0_, two parameters, fat mass (FM%) and fat-free mass (FFM%), were in the red zone, indicating severe pathological values. Furthermore, skeletal muscle mass (SMM%), total body water (TBW%), the Na^+^/K^+^-ATPase pump, and the phase angle (PhA) all fell below healthy physiological thresholds (i.e., the yellow zone), reflecting a compromised nutritional and functional status.

By 4 months (T_4_) and more markedly at 8 (T_8_) and 12 months (T_12_), a gradual shift toward normalised values (i.e., the green zone) was evident across most parameters (Figure 2).

FM% and BMI markedly decreased from 43.1% and 27.4 at T_0_ to 31.6% and 24.4 at T_12_, respectively, indicating decreased adiposity and improved metabolic health.

FFM% and SMM% increased progressively, from 56.9% to 68.4% and from 22.9% to 37.9%, respectively, transitioning from pathological to normal ranges by T_12_. This improvement likely reflects enhanced muscle anabolism and nutritional recovery, supported by dietary interventions, Qigong, and soft physical activity.

TBW%, ECW%, and ICW% also improved, suggesting better fluid distribution and cellular hydration status.

The improvement in the Na/K ratio, albeit modest, may also suggest a shift toward better electrolyte and fluid balance.

**Figure 3:**
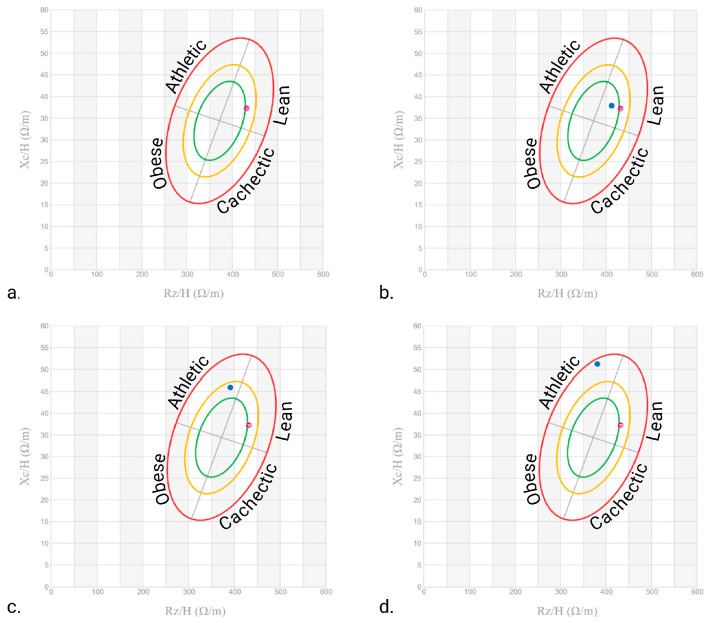
Patient bioelectrical phase angle and impedance vector analysis at (**a**) T_0_, (**b**) 4 months, (**c**) 8 months, and (**d**) 12 months. The red dots indicate the initial measurement (T_0_), whereas the blue dots indicate the follow-up measurements. The ellipses delineate the reference tolerances (50%, 75%, and 95%) for different body composition categories (Obese, Cachectic, Lean, and Athletic). Created at https://BioRender.com by Mecca, M. (accessed on 10 June 2025).

Finally, PhA increased from a low value of 4.9° to 9.0° at T_12_, which was consistent with enhanced cellular integrity and membrane function (Figure 2). As shown by the bioelectrical phase angle vector analysis (Figure 3), progressive shifts of the vector towards the “athletic” region and an increase in the PhA over time suggest improvements in muscle mass. These changes are consistent with enhanced nutritional status and physical condition. Indeed, several studies have proven that improvements in PhA are well recognised as reliable indicators of improved muscle mass, reduced inflammation, and overall better prognosis in various clinical conditions [20].

Overall, the longitudinal changes in body composition parameters, especially the increases in FFM, SMM, TBW, and PhA, support the efficacy of the integrative approach adopted, which may include dietary optimisation, potential anti-inflammatory supplementation (e.g., curcumin and polydatin), and behavioural changes.

These findings suggest a positive metabolic shift, which may be linked to the therapeutic effects of curcumin and polydatin—two bioactive compounds with well-established anti-inflammatory, antioxidant, and antitumour properties [12].

Curcumin, in particular, has been extensively studied for its role in modulating metabolic and inflammatory pathways. A meta-analysis by Mousavi et al. [38] demonstrated that curcumin supplementation significantly reduces body weight and BMI, supporting its potential as an adjunct therapy in metabolic disorders. Furthermore, both curcumin and polydatin exert antitumour effects by targeting key enzymes involved in cancer cell survival, inducing mitochondrial dysfunction, and increasing intracellular reactive oxygen species (ROS) production, ultimately promoting tumour cell apoptosis [39,40,41]. These mechanisms may explain the observed clinical improvements in our patient, particularly in the context of cancer-related metabolic dysregulation.

In addition to their direct antitumour effects, curcumin and polydatin have been shown to improve insulin sensitivity, reduce adipose tissue inflammation, and increase mitochondrial biogenesis [42,43,44,45], factors that could further contribute to patients’ improved body composition. In particular, polydatin may enhance glucose and lipid metabolism by activating the Akt signalling pathway [42] and reducing adipose tissue inflammation in preclinical models [43]. Similarly, curcumin is effective in mitigating weight gain and glucose intolerance, even after the cessation of caloric restriction [44], while promoting mitochondrial biogenesis, a key factor in metabolic health [45].

The haematological data (Table 2) support the hypothesis that the integrative approach implemented in this case maintained haematopoietic stability and supported overall physiological resilience following breast cancer surgery. The absence of significant haematologic toxicity or anaemia over 12 months is noteworthy and suggests that the combined interventions may have contributed positively to haematologic homeostasis, potentially through anti-inflammatory and antioxidant pathways.

Additionally, the structured Qigong practice—followed by self-managed gentle exercise—appears to have played a crucial role in enhancing muscle preservation and fat metabolism (Figure 3). Even after the formal Qigong sessions ended, the patient’s progress was maintained, as she continued to perform gentle gymnastics and walk outside, demonstrating the sustainability of these interventions.

In addition to physical changes, the patient reported notable improvements in chemotherapy-related pain, fatigue, and overall energy levels. Such improvements were evident from the data collected at each visit by administering the patient QoL questionnaires that combine the ESAS and SF-12 scales. The reduction in discomfort aligns with existing evidence on the analgesic effects of Qigong, possibly through its influence on stress modulation and neuromuscular relaxation [46].

These benefits may stem from the ability of Qigong to modulate stress responses and promote neuromuscular relaxation, as demonstrated in studies on persistent postsurgical pain (PPSP). For example, Osypiuk et al. [47] reported that a 12-week Qigong Mind–Body Exercise (QMBE) program led to statistically significant improvements in pain severity, fatigue, anxiety, and quality of life among breast cancer survivors, which aligns with our patient’s subjective report. In support of these observations, Xu et al. [48] reported that Qigong and Tai Chi (QTC) safely improved overall QoL in cancer patients, with notable benefits in terms of physical functioning, fatigue, sleep quality, and psychological well-being. Similarly, Qing Zeng et al. [49] highlighted Qigong’s positive effects on cognitive function and executive processing, suggesting its broader role in addressing cancer-related cognitive impairment. However, Li et al.’s meta-analysis [50] (encompassing 65 studies) demonstrated the utility of Tai Chi and Qigong in managing sleep disturbances and depression—conditions prevalent in more than 60% of postsurgical breast cancer patients. These psychological and physiological disruptions, if unaddressed, can exacerbate immune and endocrine dysfunction, potentially compromising treatment efficacy and long-term outcomes. Li et al. [50] noted that brief sessions (<60 min) could provide meaningful symptomatic relief and improvements in QoL. Moreover, the patient’s favourable outcomes may have been influenced by her consistent engagement in complementary therapies.

Equally outstanding were the psychological benefits for the patient: her mood stabilised, her sleep became more regular, and her previously reported anxiety and melancholy dissipated. These changes may be linked to both the physiological effects of curcumin and polydatin and the psychosomatic benefits of Qigong, which promotes mindfulness and stress resilience. Clinically, her visible serenity and questionnaire-reported increase in vitality speak to the profound impact of addressing both body and mind in cancer recovery.

While these findings are promising, a particular challenge lies in the multimodal intervention approach, where the simultaneous implementation of nutraceutical supplementation, Qigong practice, dietary modifications, and autonomous exercise makes it impossible to determine the individual contribution of each component to the observed outcomes.

Several limitations may arise during the implementation and analysis of studies on integrative therapies: (i) recruiting a sufficiently large and diverse sample of breast cancer (BC) patients who are willing to participate in a randomised controlled trial (RCT) and adhere to the intervention protocol; (ii) since participants in the intervention group are aware that they are receiving integrative therapy, expectation bias can occur, influencing their perceptions of improvement based on their beliefs; (iii) QoL in BC patients may fluctuate due to conventional cancer treatments (e.g., chemotherapy, radiotherapy, and surgery), which can make it challenging to isolate the specific effects of integrative oncology interventions.

To overcome these limitations and improve future studies and controlled trials, several strategies can be proposed: (a) engaging in early education campaigns to inform participants about the benefits of integrative oncology approaches; (b) developing a detailed protocol for integrative therapies, including standardised doses, treatment frequency, and session duration; (c) utilising objective measures, such as biomarkers (e.g., inflammatory haematological parameters, antioxidant panels, and urine analysis/metabolic profiles) and clinical outcomes, to complement subjective QoL assessments; (d) ensuring regular and comprehensive assessments at multiple time points (e.g., before, during, and after active treatment phases) to track trends and shifts in QoL using validated breast cancer-specific QoL measures; and (e) employing a placebo integrative therapy for the control group to mitigate expectation bias.

In fact, it should be noted that this case study is part of a broader ongoing research project involving a larger population of post-operative breast cancer women, in which we are refining a more detailed protocol that also includes haematochemical analyses and QoL questionnaires specific to BC patients. Upon reaching an adequate number of enrolled BC patients, a control group can be included whose members will receive only conventional treatments to better understand the mechanisms and standardisations of these interventions.

In conclusion, this case exemplifies how a personalised, multimodal strategy can enhance the quality of life of cancer survivors not only by restoring a sense of balance and energy but also by alleviating symptoms, enhancing functional status, and reducing the risk of adverse outcomes. It invites clinicians to consider broader therapeutic frameworks that extend beyond conventional treatments, embracing interventions that nurture both physical and emotional well-being.

## Figures and Tables

**Table 1:** Baseline demographic information and general health measures of the participant in the present study.

**Demographics**	
Age (years)	73
Gender	Female
Ethnicity	White
Smoking	No
Alcohol	No
Drugs	Bisphosphonates
**Health measures**	
Weight (kg)	64
Height (cm)	160
BMI	25.0

BMI value calculated as kg/m^2^ (weight in kg divided by the square of height in meters). BMI, body mass index.

**Table 2:** Haematochemical analysis and BIA results at follow-up for patient.

Parameter	T_0_	4 Months	8 Months	12 Months	Reference Range
*Haematochemical*					
Red blood cells	5.22	5.10	5.11	5.05	4.20–6.10 × 10^6^/µL
Haemoglobin	15.3	15.0	15.2	15.0	12–18 g/dL
Mean corpuscular volume	86.9	88.1	85.6	86.6	88–99 fL
Platelets	176	210	198	178	130–400 × 10^3^/µL
White blood cells	2.98	2.94	2.96	3.36	5.20–12.40 × 10^3^/µL
Glucose	93	91	85	91	74–109 mg/dL
Cholesterol	261	250	247	217	<200 mg/dL
AST	24	24	18	20	0–35 U/L
ALT	31	22	14	16	0–35 U/L
Gamma-GT	18	38	32	23	6–42 U/L
Calcium	10.19	10.01	10.13	10.10	8.60–10.20 mg/dL
Vitamin D	28.8	48.2	49.4	55.6	30–100 µg/L
*BIA*					
BMI	27.4	25.6	25.2	24.4	18–25
FM (%)	43.1	38.2	33.2	31.6	31.4–39.9
FFM (%)	56.9	61.8	66.8	68.4	60.1–68.6
SMM (%)	22.9	25.9	34.4	37.9	25.9–34.3
ICW (%)	52.4	53.1	57.1	56.0	47.0–58.0
ECW (%)	47.6	46.9	42.9	44.0	42.0–53.0
TBW (%)	42.0	45.5	49.0	50.1	46.0–57.0
Na/K	1.4	1.4	1.0	1.0	0.9–1.0
PhA	4.9	5.3	7.1	9.0	4.5–6.3°

## Data Availability

The original contributions presented in this study are included in the article. Further inquiries can be directed to the corresponding author.

## Abbreviations

The following abbreviations are used in this manuscript:

ACLM	American College of Lifestyle Medicine
AMICO	Ambulatorio di Medicina Integrata e Condotta in Oncologia
BC	Breast cancer
BIA	Bioelectrical impedance analysis
BMI	Body mass index
CRF	Cancer-related fatigue
ER	Oestrogen receptor
ECW	Extracellular water
FFM	Fat-free mass
FM	Fat mass
Gy	Gray
HER2	Human epidermal growth factor receptor 2
HP	Histopathological
ICW	Intracellular water
IHC	Immunohistochemical
Na^+^/K^+^ pump	Sodium-potassium pump
NST	No special type
PhA	Phase angle
PPSP	Persistent postsurgical pain
PR	Progesterone receptor
PUFAs	Polyunsaturated fatty acids
QMBE	Qigong Mind–Body Exercise
QoL	Quality of life
QTC	Qigong and Tai Chi
ROS	Reactive oxygen species
SIO	Society for Integrative Oncology
SMM	Skeletal muscle mass
TBW	Total body water
